# Ginsenoside Rg3 Serves as an Adjuvant Chemotherapeutic Agent and VEGF Inhibitor in the Treatment of Non-Small Cell Lung Cancer: A Meta-Analysis and Systematic Review

**DOI:** 10.1155/2016/7826753

**Published:** 2016-10-05

**Authors:** Tao Xu, Zhichao Jin, Yuan Yuan, Huamin Wei, Xinyao Xu, Shulin He, Shuntai Chen, Wei Hou, Qiujun Guo, Baojin Hua

**Affiliations:** ^1^Department of Oncology, Guang'anmen Hospital, China Academy of Chinese Medicine Sciences, No. 5 Beixiange, Xicheng District, Beijing 100053, China; ^2^Department of Oncology, Xiyuan Hospital, China Academy of Chinese Medicine Sciences, No. 1 Playground Road, Haidian District, Beijing 100091, China; ^3^Beijing University of Chinese Medicine, No. 11 North Third Ring Road East, Chaoyang District, Beijing 100029, China

## Abstract

*Objective.* To evaluate ginsenoside Rg3 combined with chemotherapy for non-small-cell lung cancer (NSCLC) treatment, in a meta-analysis.* Materials and Methods*. We searched PubMed, EMBASE, the Cochrane Library, the China National Knowledge Infrastructure, and the VIP and Wanfang databases for eligible studies. We manually searched for printed journals and relevant textbooks. Statistical analyses were performed with Revman 5.3 and STATA 14.0 software packages.* Results*. Twenty studies were included. Ginsenoside Rg3 combined with chemotherapy could enhance response, improve disease control, prolong overall survival, improve patient quality of life, reduce leucocyte count decrease due to chemotherapy, reduce vascular endothelial growth factor expression in peripheral blood, and increase CD4/CD8 T cell ratio.* Conclusion*. Ginsenoside Rg3 combined with chemotherapy may enhance short-term efficacy and overall survival, alleviate treatment-induced side effects, reduce vascular endothelial growth factor expression, increase CD4/CD8 T cell ratio, and serve as a potential therapeutic regimen for NSCLC. However, considering the limitations, the conclusion should be interpreted carefully, and these results need to be confirmed by more high-quality trials.

## 1. Introduction

Lung cancer is a malignant neoplasm with the highest morbidity and mortality of all tumor types. Non-small cell lung cancer (NSCLC) is the most common subtype of lung cancer and includes squamous carcinoma, adenocarcinoma, and adenosquamous carcinoma. Besides the tumor related symptoms (irritable cough, chest distress, and hemoptysis), NSCLC downregulates the CD4/CD8 ratio in patients' peripheral blood and weakens the immune system. Tumor resection is the only radical treatment with any curative potential. Many patients lose the opportunity for resection due to locally advanced and metastatic disease, although early screening for lung cancer is slowly becoming standard in many countries. Chemotherapy regimens containing platinum, taxanes, or vinorelbine are the preferred and most effective drug-related therapeutic approaches in advanced NSCLC, but they cause serious side effects such as myelosuppression, gastrointestinal reactions, alopecia, and peripheral neurotoxicity.

Traditional Chinese medicine (TCM) has been used to treat tumors for thousands of years in Eastern countries. It is accepted that TCM can inhibit tumor growth and metastasis, improve antitumor immunity, and relieve tumor-induced pain and the side effects of chemotherapy [1, 2]. Furthermore, TCM has shown a synergistic and attenuated effect when combined with chemotherapy in both basic and clinical studies [3, 4].

Ginsenoside Rg3 (Rg3) is one of the most effective steroidal saponins extracted from Ginseng, a common TCM herb which tonifies Qi in TCM theory and inhibits tumors. Rg3 suppresses tumor growth and tumor angiogenesis and endogenous vascular endothelial growth factor (VEGF) secretion by inhibiting VEGF-dependent pathways [5, 6]. Furthermore, Rg3 enhances the susceptibility of patients to chemotherapy [7, 8]. For its significant antitumor effects, Rg3 has been used in clinical trials in combination with chemotherapy regimens. For instance, Rg3 could improve the survival rate in advanced gastric cancer patients and when combined with adjuvant chemotherapy [9]. It is also indicated that Rg3, especially in combination with chemotherapy, can improve the life span of patients with NSCLC after operation [10]. Based on that, we conducted a systematic review and meta-analysis to evaluate the effects of Rg3 on NSCLC treatment. This study was conducted according to the PRISMA guidelines (Supplementary File 4, in Supplementary Material available online at http://dx.doi.org/10.1155/2016/7826753) [11, 12].

## 2. Materials and Methods

### 2.1. Literature Search

Studies were explored from databases including PubMed (from Jan 1975 to Aug 2016), the Cochrane Library (from Jan 2010 to Aug 2016), Excerpta Medica database (EMBASE) (from Jan 1990 to Aug 2016), China National Knowledge Infrastructure (CNKI) (from Jan 1979 to Aug 2016), Weipu database (VIP) (from Jan 1990 to Aug 2016), and Wanfang database (WF) (from Jan 1989 to Aug 2016). All the studies were searched regardless of their publication type and without language restriction. Key words, MESH terms, and search strategies for each database were as follows.

### 2.2. (Rg3 or Ginsenoside Rg3) and (Lung Cancer or Lung Tumor or Non-Small Cell Lung Cancer)

In addition to electronic databases, printed journals and relevant textbooks were manually searched from the libraries of Beijing University of Chinese Medicine, Peking Union Medical College, and Guang'anmen Hospital. Specialized experts in particular fields were also consulted for necessary supplements.

Inclusion criteria are as follows: (1) types of studies: randomized clinical trials (RCTs); (2) participants: adult human populations (over 18 years of age) who were pathologically diagnosed with clinical stage III (unresectable) and IV NSCLC; (3) interventions: the control group treated with chemotherapy and the experiment group treated with the same chemotherapeutic regimens plus Rg3; (4) outcomes: short/long-term chemotherapy response rate, Karnofsky's performance score (KPS), chemotherapeutic side effects such as myelosuppression and gastrointestinal symptoms, pain management, immunity index, and VEGF levels in the peripheral blood.

Exclusion criteria are as follows: (1) studies such as reviews, animal research, observational studies without a control group, or other types of non-RCT studies; (2) trials about other types of tumors or small-cell lung cancer; (3) participants who had nonpathological diagnosis, previously subjected to chemotherapy, radiotherapy, or surgery, concurrent infection, other malignancies, or serious medical illnesses; (4) participants in control group treated with other antitumor TCM drugs.

### 2.3. Literature Selection and Data Extraction

Two independent reviewers (Yuan Yuan and Zhichao Jin) evaluated each title, abstract, and citation and selected relevant studies according to the inclusion criteria. Duplicates identification strategy was as follows: “type-I” (“duplicates among/across different databases”) and “type-II” (duplicate publications in different journals/issues) identified by a pragmatic strategy of combining auto- and hand-searching methods [33]. Disagreements were discussed with and resolved by a third reviewer (Wei Hou). Data from included studies were extracted separately by Xinyao Xu and Shulin He by using a specific form and checked by Shuntai Chen. The characteristics of the data included name of first author, year of publication, sex, and number of cases and controls, methods of randomization, interventions, treatment periods, and outcomes. The hazard ratio (HR) was calculated from the Kaplan-Meier survival curve and survival outcome events as reported by Tierney et al. [34].

### 2.4. Quality Assessment of Studies

The methodological quality of each RCT was independently assessed by Tao Xu and Huamin Wei using the Cochrane Risk of Bias tool. Disagreements were discussed with and resolved by Baojin Hua.

### 2.5. Data Synthesis and Analysis

Statistical analyses were performed using Review Manager (RevMan) 5.3.5 software (Cochrane Community, London, UK) and STATA 14 software. The total effectiveness rates of dichotomous data were pooled using risk ratios (RRs) with 95% confidence interval (CI). *P* < 0.05 was considered statistically significant. The heterogeneity of the included studies was evaluated by the *χ* ^2^ and *I* ^2^ tests, and *P* < 0.10 or *I* ^2^ > 50% was defined as indicating heterogeneity. The fixed-effect model was used in homogeneity data merging and the random-effects model was suitable for the merging of heterogeneous data. Publication bias was evaluated by visual assessment of the asymmetry of funnel plots (RevMan 5.3.5) and Egger's test (STATA 14) with *P* < 0.05 indicating potential bias. Sensitivity analysis was evaluated by reanalyzing the data using different statistical approaches.

## 3. Results

### 3.1. Eligible Studies

**Figure 1:**
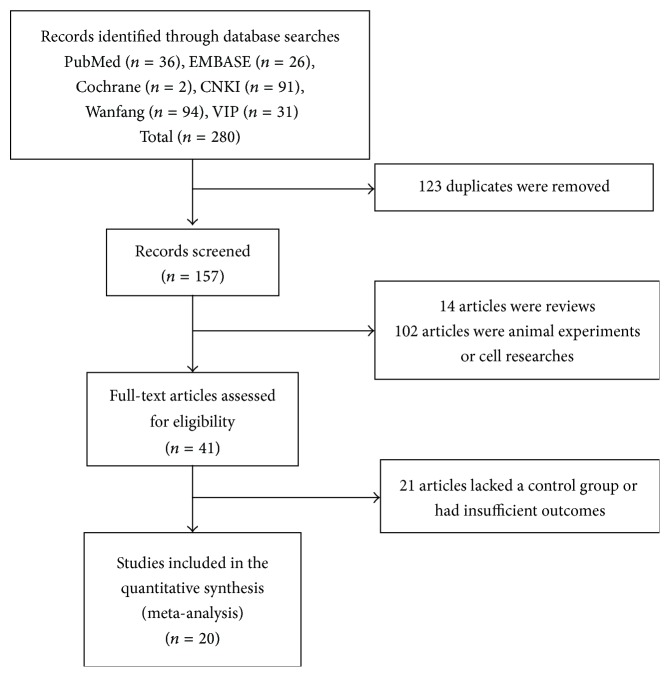
Flow diagram of the literature search process.

A total of 280 studies were found during the initial search, among which 123 duplicated studies were removed along with another 116 studies that met one or more of the exclusion criteria. After reading the full text, another 21 studies were excluded because they lacked a control group or had insufficient outcomes. Ultimately, 20 studies were included in the final analysis (Figure 1).

#### 3.1.1. Study Characteristics

Twenty studies with a total of 1315 patients were included, with 671 subjects in the experimental groups and 644 in the control groups. Characteristics such as sample size, sex, age, interventions, and outcomes for each study are described in Table 1.

#### 3.1.2. Quality Assessment

**Figure 2:**
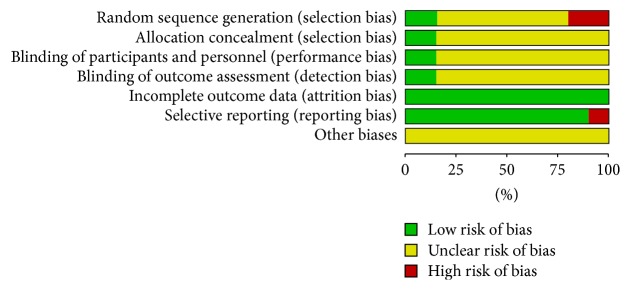
Risk of bias graph: review of authors' judgments about each risk of bias presented as percentages across all included studies.

**Figure 3:**
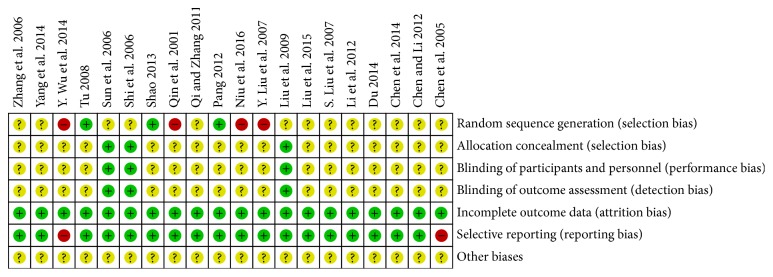
Risk of bias summary: review of authors' judgments about each risk of bias for each included study.

All of the included studies applied randomization, but 13 of them did not describe the randomization method in detail and four of them had a high risk of bias because the sequence was generated by the date of admission or the condition of the patients. All the included studies had complete data but only three of them mentioned the details of allocation concealment and blinding of participants and personnel and outcome assessment. Two studies had a high risk of reporting bias for one or more outcomes; also, the data for some of the outcomes were reported incompletely so they could not be entered in a meta-analysis (Table 2, Figures 2 and 3).

### 3.2. Rg3 and Response Rate

**Figure 4:**
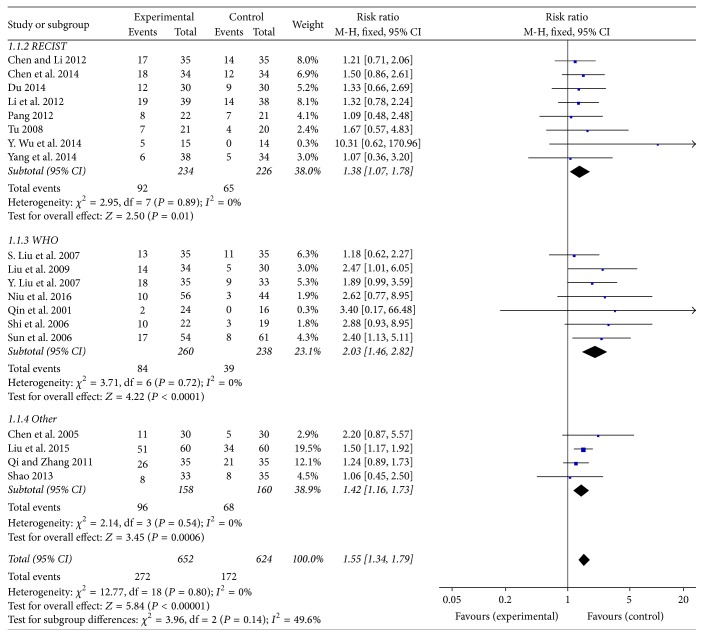
Forest plot of risk ratio (RR) for evaluating the chemotherapy response rate in a fixed-effect model. The RR of chemotherapy response rate in Rg3 and chemotherapy group was compared with that of the chemotherapy group. Individual study is shown by the square with blue color, and the pooled datasets were shown by the diamond, representing the 95% confidence interval (CI) of each study. RR > 1 implied a better chemotherapy response rate of the experimental group. The size of each investigation represented the weighting factor (1/SE) assigned to the study.

Rg3 may enhance the response rate to chemotherapy in NSCLC patients. Nineteen studies evaluated the response rate to chemotherapy. The response rate in the experiment group (Rg3 combined with chemotherapy) was significantly higher than that in the control group (chemotherapy only) (RR = 1.55, 95% CI: 1.34–1.79, and *P* < 0.00001 in the *Z* test). The result did not indicate the heterogeneity with *χ* ^2^ = 12.77, *P* = 0.80, and *I* ^2^ = 0%. Subgroups were divided by different evaluation criteria: 8 studies followed the Response Evaluation Criteria in Solid Tumors (RECIST) guidelines, 6 studies followed WHO guidelines, and 4 studies followed other guidelines. There was no significant difference between three subgroups (*P* = 0.14), and evaluations of the three showed the same result (Figure 4).

### 3.3. Rg3 and the Disease Control Rate

**Figure 5:**
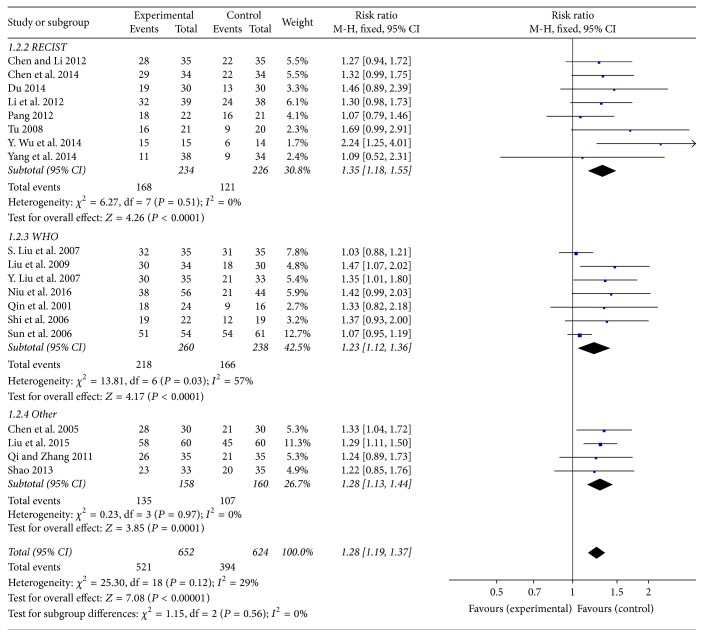
Forest plot of RR for evaluating the disease control rate in a fixed-effects model. The RR of disease control rate in the Rg3 and chemotherapy group was compared with that of the chemotherapy group. Individual studies are shown by the blue-colored squares, and the pooled datasets are shown by the diamond, representing the 95% confidence interval (CI) of each study. RR > 1 implied a better disease control rate of the experimental group. The size of each investigation represented the weighting factor (1/SE) assigned to the study.

Rg3 may enhance the disease control rate when combined with chemotherapy in NSCLC. Nineteen studies evaluated the disease control rate of chemotherapy. The disease control rate in the experimental group was significantly higher than that in the control group (RR = 1.28, 95% CI: 1.19–1.37, and *P* < 0.00001 in the *Z* test). The result did not indicate much heterogeneity with *χ* ^2^ = 23.99, *P* = 0.12, and *I* ^2^ = 29%. Subgroups were divided as mentioned above: 8 studies followed RECIST guidelines, 6 studies followed WHO guidelines, and 4 studies followed other guidelines. There was no significant difference between the three subgroups (*P* = 0.56), and evaluations of the three showed the same result (Figure 5).

### 3.4. Rg3 Prolonged Overall Survival following Chemotherapy

**Figure 6:**
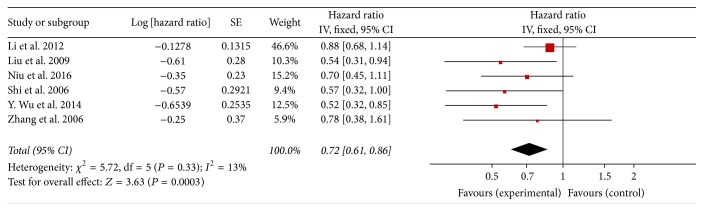
Forest plot of (hazard ratio) HR for evaluation of overall survival in fixed-effect model. The HR of overall survival in Rg3 and chemotherapy group was compared with that of the chemotherapy group. Individual studies are shown by the red-colored squares, and the pooled datasets are shown by the diamond, representing the 95% confidence interval (CI) of each study. HR < 1 implied improved overall survival in the experimental group. The size of each investigation represented the weighting factor (1/SE) assigned to the study.

Six studies compared long-term survival between the experimental and control groups. The pooled hazard ratio (HR) was 0.72, 95% CI was 0.61–0.86, and *P* = 0.0003 in *Z* test. The heterogeneity was not significant (*P* = 0.33, *I* ^2^ = 13%) (Figure 6).

### 3.5. Rg3 Improved Quality of Life for Late-Stage NSCLC Patients

**Figure 7:**
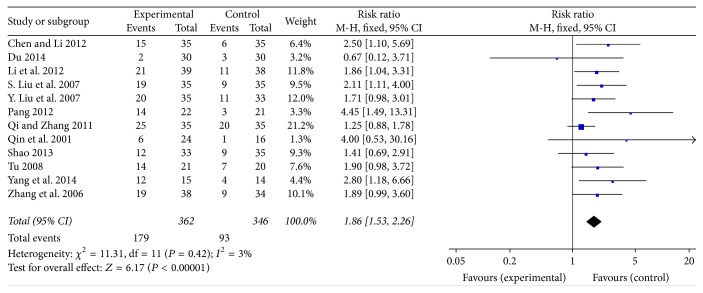
Forest plot of RR for evaluation of KPS in late-stage NSCLC patients in a fixed-effect model. The RR of KPS in the Rg3 and chemotherapy group was compared with that of the chemotherapy group. Individual studies are shown by the blue-colored squares, and the pooled datasets are shown by the diamond, representing the 95% confidence interval (CI) of each study. RR > 1 implied a better quality of life in late-stage NSCLC patients among the experimental group. The size of each investigation represented the weighting factor (1/SE) assigned to the study.

The improvement of KPS was pooled for evaluation and the RR was 1.86, 95% CI was 1.53–2.26, and *P* < 0.00001. The result did not indicate the heterogeneity (*χ* ^2^ = 11.31, df = 11, *P* = 0.42, and *I* ^2^ = 3%) (Figure 7).

### 3.6. Rg3 May Reduce the Decline of Leucocyte Count due to Chemotherapy

**Figure 8:**
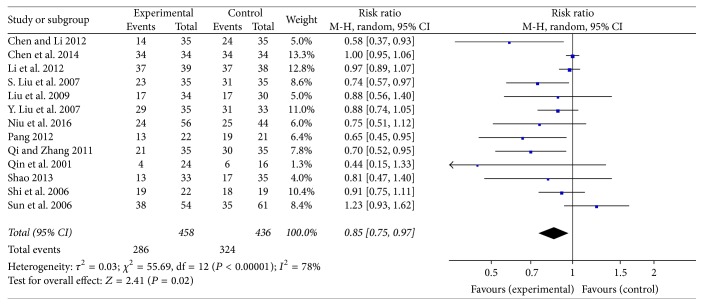
Forest plot of RR evaluating the decline in leucocyte count in a random-effect model. The RR in the Rg3 and chemotherapy group was compared with that of the chemotherapy group. Individual studies are shown by the blue-colored squares, and the pooled datasets are shown by the diamond, representing the 95% confidence interval (CI) of each study. RR > 1 implied a lower decline of leucocyte count in the experimental group. The size of each investigation represented the weighting factor (1/SE) assigned to the study.

Thirteen studies evaluated leukocyte counts among NSCLC patients between experimental and control groups (pooled RR = 0.85, 95% CI = 0.75–0.97) (*P* = 0.02). There was significant heterogeneity (*P* < 0.00001, *I* ^2^ = 78%), so we used the random-effects model (Figure 8).

### 3.7. Rg3 Could Reduce the Expression of VEGF in Peripheral Blood

**Figure 9:**
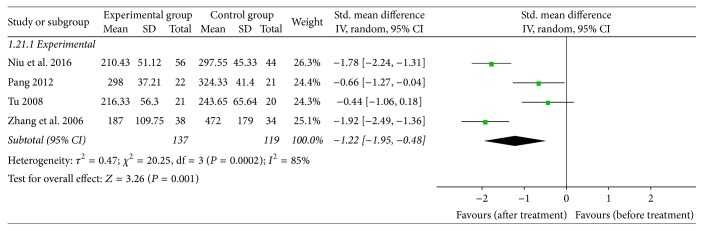
Forest plot of the Std. mean difference (SMD) for evaluating VEGF expression in peripheral blood of NSCLC patients at the periods before and after treatment in a random-effect model. The SMD of expression of VEGF in peripheral blood in the Rg3 and chemotherapy group was compared with that of the chemotherapy group. Individual studies are shown by the green-colored squares, and the pooled datasets are shown by the diamond, representing the 95% confidence interval (CI) of each study. SMD < 0 and *P* < 0.05 implied a lower expression of VEGF in the experimental group. The size of each investigation represented the weighting factor (1/SE) assigned to the study.

Four studies compared VEGF expressions in the peripheral blood of NSCLC patients before and after treatment. Results indicated that VEGF expression was significantly reduced after treatment in the experimental group compared to the control group (Std. mean difference = −1.22, 95% CI = −1.95 to −0.48). There was a significant heterogeneity between the two groups (*I* ^2^ = 85%, *P* = 0.0002), so we used the random-effect model (Figure 9).

### 3.8. Rg3 Could Enhance the Ratio of CD4/CD8

**Figure 10:**
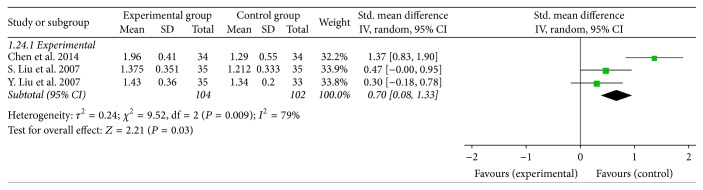
Forest plot of Std. mean difference (SMD) for evaluating the ratio of CD4/CD8 in the peripheral blood of NSCLC patients at the periods before and after treatment in a random-effect model. The SMD of the ratio of CD4/CD8 in the peripheral blood in the Rg3 and chemotherapy group was compared with that of the chemotherapy group. Individual studies are shown by the green-colored squares, and the pooled datasets are shown by the diamonds, representing the 95% confidence interval (CI) of each study. SMD > 0 and *P* < 0.05 implied a more enhancement of the ratio of CD4/CD8 in experimental group. The size of each investigation represented the weighting factor (1/SE) assigned to the study.

Three studies compared the ratios of CD4/CD8 in peripheral blood of NSCLC patients before and after treatment. The result indicated that the ratio of CD4/CD8 was significantly enhanced after Rg3 treatment in the experimental group (Std. mean difference = 0.70, 95% CI = −0.08 to 1.33). As there was a heterogeneity between the two groups (*I* ^2^ = 79%, *P* = 0.009), we used the random-effect model (Figure 10).

### 3.9. Other Negative Results

However, 11 studies evaluated the incidence of anemia induced by chemotherapy in the random-effect model (*I* ^2^ = 59%, *P* = 0.006), and the results showed that Rg3 could not alleviate chemotherapy-induced anemia (RR = 0.84, 95% CI = 0.67–1.06, and *P* = 0.14) (Figure 1, Supplementary File 2). The results of the 11 studies according to the random-effect model (*I* ^2^ = 63%, *P* = 0.002) demonstrated that Rg3 could not reduce the declination of platelet count due to chemotherapy (RR = 0.87, 95% CI = 0.71–1.07, and *P* = 0.19) (Figure 2, Supplementary File 2). In addition, Rg3 had no significant effect on digestive reactions such as nausea and vomiting or constipation, with 11 and 2 studies evaluating these aspects, respectively (RR = 0.97, 95% CI = 0.88–1.07, and *P* = 0.53; RR = 0.71, 95% CI = 0.40–1.28, and *P* = 0.26; Figures 3 and 4, Supplementary File 2). Some other side effects such as hepatic dysfunction, peripheral nerve toxicity, alopecia, and fatigue induced by chemotherapy could not be improved by Rg3 (RR = 0.99, 95% CI = 0.66–1.49, and *P* = 0.97; RR = 1.34, 95% CI = 0.45–3.95, and *P* = 0.60; RR = 0.92, 95% CI = 0.57–1.49, and *P* = 0.74; RR = 1.08, 95% CI = 0.42–2.78, and *P* = 0.87) (Figures 5, 6, 7, and 8, Supplementary File 2).

### 3.10. Sensitivity Analysis

Results of the sensitivity analyses showed that changing the study effect model did not change the results of the pooled analyses (Table 3, Supplementary File 1).

### 3.11. Publication Bias

Egger's test is based on a linear regression of the standard normal deviate against its precision. In our analysis, we used the inverse of the standard error as the independent variable and the standardized estimate of the size effect (log RR upon its standard error) as the dependent variable. The estimate of the effect is considered biased if the intercept is significantly different from zero. The test results are shown in Table 4. Therefore, Egger's tests suggested that publication bias may have a significant influence on the results of response rate, KPS, decline of platelet count, and hepatic dysfunction (Table 4, Supplementary File 3).

## 4. Discussion

Incidence and mortality rates in lung cancer are high. Although targeted therapies such as EGFR tyrosine kinase inhibitors (TKIs) and angiogenesis inhibitors offer longer survival times in advanced NSCLC patients [35, 36], conventional chemotherapy remains the most common treatment for patients with advanced disease, with platinum-based chemotherapy regimens as first-line treatment in this patient population. These regimens combine cisplatin or carboplatin with cytotoxic drugs such as paclitaxel, docetaxel, gemcitabine, vinorelbine, and pemetrexed [37]. Cyclophosphamide [38], S-1 [39], and etoposide [40] are also treatment approaches for advanced NSCLC. However, many factors affect the curative potential of chemotherapy. It has been reported that high expression levels of ERCC1 and RRM1 may reduce the response rate and survival rate in lung cancer patients treated with cisplatin and/or gemcitabine [41]. Studies have also indicated that the expression of* TYMS*,* TUBB3*, nonmuscle myosin II, myoglobin, and* MyoD1* may also influence the curative potential of platinum-based chemotherapy [42]. In the present study, we found that Rg3 may enhance the response rate and disease control rate when combined with chemotherapy. Although there was no evidence indicating a relationship between Rg3 and any of the drug resistance genes mentioned above, researches have shown that Rg3 can inhibit the growth of lung cancer cells and prevent angiogenesis and epithelial-mesenchymal transition (EMT) and invasion of lung cancer [5, 43]. Results from a randomized, prospective, multicenter clinical trial of an NP regimen plus Rg3 illustrated the effects of Rg3 on advanced NSCLC patients in the form of improved response rates and survival times [22]. Furthermore, our analysis indicated Rg3 may improve the KPS in NSCLC patients, thereby indicating an improved quality of life.

Chemotherapy often results in side effects such as bone marrow suppression and gastrointestinal reactions. Our results showed that Rg3 may reduce the incidence of leukopenia during or after chemotherapy, but Rg3 was unable to improve myelosuppressive effects in other cells or gastrointestinal reactions. In addition, liver dysfunction, peripheral nerve toxic reaction, alopecia, and fatigue were not improved or relieved with the addition of Rg3.

The immunosuppressive microenvironment limits tumor treatment [44]. Determining lymphocyte subgroups in the peripheral blood is an effective assessment method about the immune function, and CD4^+^/CD8^+^ level decreased after several cycles of chemotherapy. Moreover, the decreasing ratio of CD4^+^/CD8^+^ was associated with tumor progression [45]. Rg3 was extracted from Ginseng, a tonic herb that can enhance immunity [46, 47]. Similarly, our results showed that Rg3 could significantly enhance the ratio of CD4^+^/CD8^+^ during chemotherapy in NSCLC patients (*P* < 0.0001). Basic researches also indicated Rg3 could enhance antitumor cellular immunity [48].

Angiogenesis is a hallmark of cancer that is a critical component of cancer progression, facilitating rapid tumor growth and metastasis [49]. VEGF is one of the main mediators of angiogenesis in NSCLC [50]. Thus, treatment with anti-angiogenesis inhibitors or those targeting the anti-VEGF pathway is an optional method in lung cancer therapy [51]. According to our analysis, Rg3 could significantly reduce the VEGF expression in NSCLC patients' peripheral blood (*P* = 0.001). Rg3 attenuated VEGF overexpression in tumor xenograft models as well [52].

This meta-analysis has some limitations. First, all included trials were first published in Chinese, resulting in low-quality papers, and publication bias was evident in some results. Second, the randomization and concealment allocation of most studies were not clear, resulting in possible bias and overestimation of efficacy. Third, the study periods were generally short, and none of the included trials included long-term follow-up. Since NSCLC has been seen as a chronic condition, the long-term effects of treatment are a major concern. Thus, designing RCTs of Rg3 plus chemotherapy to include longer follow-up times is necessary. However, although problems persist, which prevent us from drawing definite conclusion about the efficacy of Rg3, our results still provide helpful information for clinicians indicating that Rg3 can enhance drug efficacy and reduce drug-induced toxicity from chemotherapy. Well-designed clinical trials are needed to clarify the precise role of Rg3 in this treatment setting.

## 5. Conclusion

In conclusion, this meta-analysis indicated that Rg3 may enhance response rates, improve disease control rates, prolong overall survival after chemotherapy, promote an improved quality of life, reduce the treatment-related decline in leucocyte counts, reduce VEGF expression in the peripheral blood, and increase the ratio of CD4/CD8 T cells when combined with systematic chemotherapy for NSCLC. However, considering the limitations, the conclusion should be interpreted carefully, and these results need to be confirmed by more high-quality trials.

## Supplementary Material

Supplementary file1: Sensitivity analysis; Supplementary file 2: Negative results; Supplementary file 3: Egger tests; Supplementary file 4: PRISMA 2009 Checklist.

## Figures and Tables

**Table 1:** Characteristics of the included studies.

Reference	Year	Sample size (E/C)	Sex	Age (y) (E/C)	Clinical stage (E/C)	Pathology	Experimental group (E)	Control group (C)	Period	Outcome measure
Chen et al. [13]	2005	60 (30/30)	F: 25, M: 5/F: 21, M: 9	54 ± 4/59 ± 2	III: 21, IV: 9/III: 17, IV: 13	S22, A8/S19, A11	Rg3 20 mg po. Bid + C	EP (VP-16 + DDP), MVP (MMC + VDS + DDP)	6–8 weeks	Tumor response (UICC) Side effects KPS

Chen and Li [14]	2012	70 (35/35)	F: 24, M: 11/F: 22, M: 13	55.5/60.5 (average age)	III: 14, IV: 21/III: 13, IV: 22	S20, A15/S18, A17	Rg3 20 mg po. Bid + C	GP (GEM + DDP)	6–9 weeks	Tumor response (RECIST) Side effects KPS

Chen et al. [15]	2014	68 (34/34)	M: 39, F: 29	41–73 (median age 55)	III, IV	S21, A26, AS18, L3	Rg3 20 mg po. Bid + C	TP (PTX + DDP)	12 weeks	Tumor response (RECIST) Side effects Immunity

Du [16]	2014	60 (30/30)	F: 31, M: 29	35–67 (average age 40.2 ± 3.6)	IV	Non-small cell cancer	Rg3 20 mg po. Bid + C	TP (PTX + DDP)	6 weeks	Tumor response (RECIST) KPS

Li et al. [17]	2012	77 (39/38)	Unclear	Unclear	IV	S14, A23, L2/S16, A20, L2	Rg3 20 mg po. Bid + C	GP (GEM + DDP)	6 weeks	Tumor response (RECIST) Side effects KPS Median survival time 1-year survival rates

Liu et al. [18]	2007	70 (35/35)	F: 43, M: 27	35–70 (median age 56)	IV	S26, A40, L4	Rg3 20 mg po. Bid + C	NP (NVB + DDP)	6 weeks	Tumor response (WHO) Side effects KPS Median survival time Immunity

Liu et al. [19]	2015	120 (60/60)	F: 46, M: 14/F: 35, M: 25	34–71 (52.5 ± 2)/35–74 (54.6 ± 2.1) (average age)	III: 37, IV: 23/III: 29, IV: 31	S41, A19/S46, A14	Rg3 20 mg po. Bid + C	NP (NVB + DDP)	6 weeks	Tumor response (unclear) Side effects

Liu et al. [20]	2009	64 (34/30)	F: 26, M: 8/F: 19, M: 11	43–75 (62)/31–66 (58) (median age)	III: 22, IV: 12/III: 26, IV: 4	S9, A21, AS4/S6, A21, AS2	Rg3 20 mg po. Bid + C	NP (NVB + DDP)	6 weeks	Tumor response (WHO) Side effects KPS Median survival time Immunity

Liu et al. [21]	2007	68 (35/33)	F: 24, M: 11/F: 23, M: 10	65–75 (69)/65–75 (70) (median age)	IIIb: 28, IV: 7/IIIb: 23, IV: 10	S18, A15, AS2/S17, A15, AS1	Rg3 20 mg po. Bid + C	NP (NVB + DDP)	6 weeks	Tumor response (WHO) Side effects KPS Immunity

Pang [22]	2012	43 (22/21)	F: 13, M: 9/F: 13, M: 8	47–80 (average age 63.95)	III: 13, IV: 30	A26, S18	Rg3 20 mg po. Bid + C	GP, TP (DTX + DDP), PC (PEM + DDP)	6 weeks	Tumor response (RECIST) Side effects KPS VEGF∖bFGF

Qi and Zhang [23]	2011	70 (35/35)	M: 48, F: 22	Median age 57	IV	S26, A40, L4	Rg3 20 mg po. Bid + C	NP (NVB + DDP)	12 weeks	Tumor response (unclear) Side effects KPS

Qin et al. [24]	2001	39 (23/16)	F: 19, M: 4/F: 13, M: 3	Median age 59.6/57.2	III: 18, IV: 5/III: 12, IV: 4	S11, A8, AS4/S7, A6, AS3	Rg3 20 mg po. Bid + C	EP (VP-16 + DDP)	8 weeks	Tumor response (WHO) Side effects KPS

Shao [25]	2013	68 (33/35)	F: 23, M: 10/F: 26, M: 9	65–80 (71 ± 4)/65–81 (72 ± 4) (average age)	III: 26, IV: 7/III: 26, IV: 9	S15, A17, P1/S17, A16, P2	Rg3 20 mg po. Bid + C	DTX	6 weeks	Tumor response (unclear) Side effects KPS

Shi et al. [26]	2006	41 (22/19)	F: 16, M: 6/F: 15, M: 4	45–75 (62)/37–64 (58) (median age)	III: 5, IV: 17/III: 5, IV: 14	S9, A12, AS1/S4, A14, P1	Rg3 20 mg po. Bid + C	NP (NVB + DDP), MVP (MMC + VDS + DDP)	24 weeks	Tumor response (WHO) Side effects Median survival time 1/2-year survival rates

Sun et al. [27]	2006	115 (54/61)	M: 40, F: 14/M: 39, F: 22	22–75 (62)/32–74 (62) (median age)	III: 21, IV: 33/III: 24/IV: 37	S16, A27, AS6/S13, A44, AS2	Rg3 20 mg po. Bid + C	NP (NVB + DDP)	6 weeks	Tumor response (WHO) Side effects KPS Median survival time

Tu [28]	2008	41 (21/20)	M: 13, F: 8/M: 11, F: 9	36–75 (56.7) (average age)	III: 7, IV: 14/III: 8, IV: 12	S7, A10, O4/S9, A9, O2	Rg3 20 mg po. Bid + C	TP (PTX + DDP)	At least 6 weeks	Tumor response (RECIST) KPS VEGF

Wu et al. [29]	2014	40 (20/20)	M: 11, F: 9/M: 11, F: 9	47–77 (60.6)/45–83 (62.2) (median age)	III: 11, IV: 9/III: 11, IV: 9	S6, A12, AS2/S6, A13, AS1	Rg3 20 mg po. Bid + C	GP/NP/TP	12–18 weeks	Tumor response (RECIST) Side effects TTP/OS

Yang et al. [30]	2014	29 (15/14)	M: 11, F: 4/M: 10, F: 4	70–85 (76) (average age)	III: 9, IV: 6/III: 7, IV: 7	S5, A9/S4, A10	Rg3 20 mg po. Bid + C	S-1	12 weeks	Tumor response (RECIST) Side effects KPS

Zhang et al. [31]	2006	72 (38/34)	Unclear	53.2/51.9 (median age)	III: 23, IV: 15/III: 21, IV: 13	S19, A15, AS4/S17, A14, AS3	Rg3 20 mg po. Bid + C	CTX	12 weeks	KPS VEGF Immunity TTP Median survival time 1-year survival rates

Niu et al. [32]	2016	100 (56/44)	M: 68, F: 32	38–72 (average age 53.12 ± 4.75)	IV	Non-small cell cancer	Rg3 20 mg po. Bid + C	PTX	12 weeks	Tumor response (WHO) Side effects VEGF

S: squamous carcinoma; A: adenocarcinoma; AS: adenosquamous carcinoma; L: large cell carcinoma; P: poorly differentiated; O: other types; VP-16: etoposide; DDP: cisplatin; MMC: mitomycin; VDS: vindesine; GEM: gemcitabine; PTX: paclitaxel; NVB: vinorelbine; DTX: docetaxel; PEM: pemetrexed; S-1: tegafur gimeracil oteracil potassium capsule; CTX: cyclophosphamide; E/C: experiment group/control group.

**Table 2:** Quality assessment of the included studies.

Trials	Randomization	Concealment allocation	Blinding of participants	Blinding of outcome assessors	Incomplete outcome data	Selective reporting	Other sources of bias
Chen et al. [13]	Unclear	Unclear	Unclear	Unclear	Low risk	High risk^c^	Unclear
Chen and Li [14]	Unclear	Unclear	Unclear	Unclear	Low risk	Low risk	Unclear
Chen et al. [15]	Unclear	Unclear	Unclear	Unclear	Low risk	Low risk	Unclear
Du [16]	Unclear	Unclear	Unclear	Unclear	Low risk	Low risk	Unclear
Li et al. [17]	Unclear	Unclear	Unclear	Unclear	Low risk	Low risk	Unclear
Liu et al. [18]	Unclear	Unclear	Unclear	Unclear	Low risk	Low risk	Unclear
Liu et al. [19]	Unclear	Unclear	Unclear	Unclear	Low risk	Low risk	Unclear
Liu et al. [20]	Unclear	Low risk	Low risk	Low risk	Low risk	Low risk	Unclear
Liu et al. [21]	High risk^a^	Unclear	Unclear	Unclear	Low risk	Low risk	Unclear
Pang [22]	Low risk^b^	Unclear	Unclear	Unclear	Low risk	Low risk	Unclear
Qi and Zhang [23]	Unclear	Unclear	Unclear	Unclear	Low risk	Low risk	Unclear
Qin et al. [24]	High risk^a^	Unclear	Unclear	Unclear	Low risk	Low risk	Unclear
Shao [25]	Low risk^b^	Unclear	Unclear	Unclear	Low risk	Low risk	Unclear
Shi et al. [26]	Unclear	Low risk	Low risk	Low risk	Low risk	Low risk	Unclear
Sun et al. [27]	Unclear	Low risk	Low risk	Low risk	Low risk	Low risk	Unclear
Tu [28]	Low risk^b^	Unclear	Unclear	Unclear	Low risk	Low risk	Unclear
Wu et al. [29]	High risk^a^	Unclear	Unclear	Unclear	Low risk	High risk^c^	Unclear
Yang et al. [30]	Unclear	Unclear	Unclear	Unclear	Low risk	Low risk	Unclear
Zhang et al. [31]	Unclear	Unclear	Unclear	Unclear	Low risk	Low risk	Unclear
Niu et al. [32]	High risk^a^	Unclear	Unclear	Unclear	Low risk	Low risk	Unclear

^a^Sequence generated by the date of admission or the condition of patients.

^b^Referring to a random number table.

^c^One or more outcomes of interest in the review are reported incompletely so that they cannot be entered in a meta-analysis.

**Table 3:** Sensitivity analysis.

	Number of studies	Results [95% CI]		Heterogeneity		Effect measure
		Fixed-effect model	Random-effect model	*I* ^2^ (%)	*P* value	
Response rate	18	1.55 [1.34, 1.79]	1.47 [1.28, 1.68]	0	0.80	Risk ratio
Disease control rate	18	1.28 [1.19, 1.37]	1.25 [1.15, 1.35]	29	0.12	Risk ratio
Overall survival	5	0.72 [0.61, 0.86]	0.70 [0.58, 0.86]	13	0.33	Hazard ratio
KPS	12	1.86 [1.53, 2.26]	1.74 [1.43, 2.12]	3	0.42	Risk ratio
Decline of leucocyte count	12	0.85 [0.79, 0.92]	0.85 [0.75, 0.97]	78	<0.00001	Risk ratio
VEGF	4	−1.32 [−1.59, −0.04]	−1.22 [−1.95, −0.48]	85	0.0002	SMD
Ratio of CD4/CD8	3	0.67 [0.38, 0.95]	0.70 [0.08, 1.33]	79	0.009	SMD

**Table 4:** Publication bias.

Egger's publication test	*P* value
Response rate	0.035
Disease control rate	0.455
KPS	0.046
Decline of leucocyte count	0.448
Anemia	0.182
Decline of platelet count	0.029
Nausea and vomiting	0.159
Hepatic dysfunction	0.023
Alopecia	0.285
Overall survival	0.083
