# Supplementary material for: Ginsenoside Rg3 Serves as an Adjuvant Chemotherapeutic Agent and VEGF Inhibitor in the Treatment of Non-Small Cell Lung Cancer: A Meta-Analysis and Systematic Review
Source: Evid Based Complement Alternat Med. 2016 Oct 5;2016:7826753. doi: 10.1155/2016/7826753 (PMC5069366; doi:10.1155/2016/7826753)
Supplement: Supplementary file 1 — Supplementary file1: Sensitivity analysis; Supplementary file 2: Negative results; Supplementary file 3: Egger tests; Supplementary file 4: PRISMA 2009 Checklist. [file 7826753.f1.docx]

**Supplementary file 1: Sensitivity analysis**

**
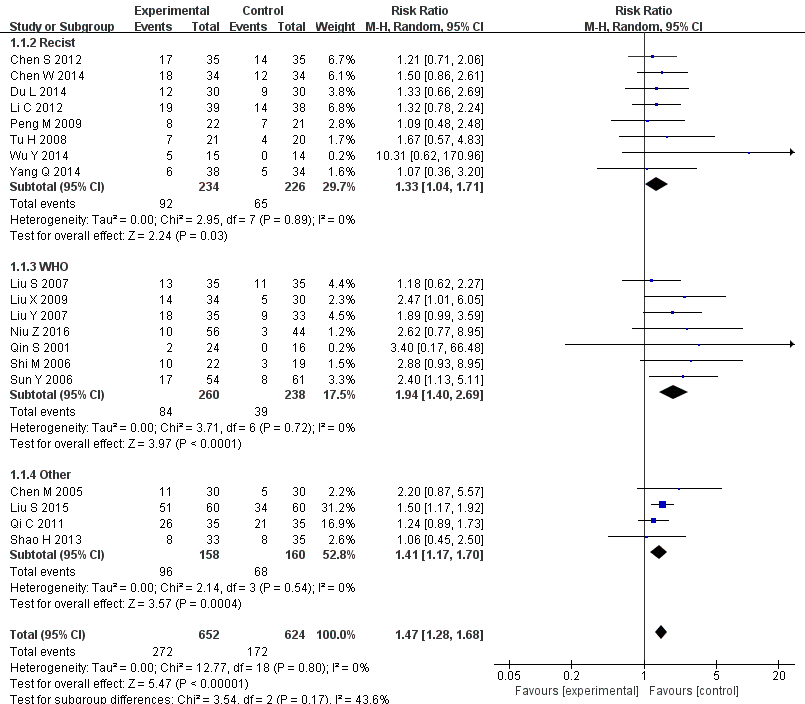
**

**Figure 1. Forrest plot of risk ratio (RR) for evaluating the chemotherapy response rate in a random effect model.** The RR of chemotherapy response rate in Rg3 and chemotherapy group was compared with the chemotherapy group. Individual study is shown in the square with blue color, and the pooled datasets were shown in the diamond, representing the 95% conﬁdence interval (CI) of each study. RR > 1 implied a better chemotherapy response rate of the experimental group. The size of each investigation represented the weighting factor (1/SE) assigned to the study.


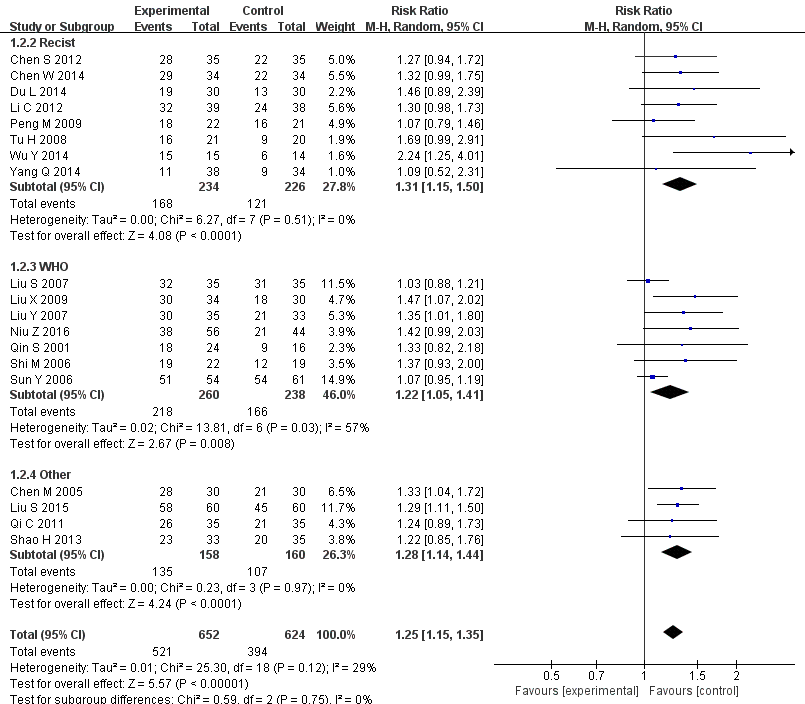


**Figure2: Forest plot of RR for evaluating the disease control rate in a random effect model.** The RR of disease control rate in the Rg3 and chemotherapy group was compared with the chemotherapy group. Individual studies are shown in the blue-colored squares, and the pooled datasets are shown by the diamond, representing the 95% conﬁdence interval (CI) of each study. RR > 1 implied a better disease control rate of the experimental group. The size of each investigation represented the weighting factor (1/SE) assigned to the study.


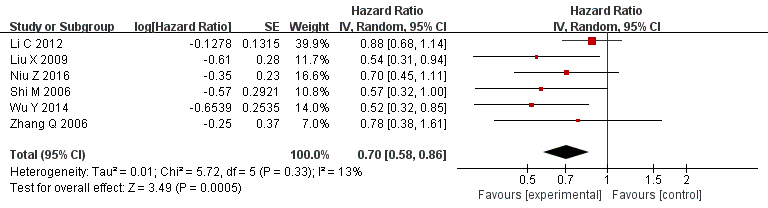


**Figure3: Forest plot of (Hazard Ratio) HR for evaluating of overall survival in a random effects model.** The HR of overall survival in Rg3 and chemotherapy group was compared with the chemotherapy group. Individual studies are shown in the red-colored squares, and the pooled datasets are shown by the diamond, representing the 95% conﬁdence interval (CI) of each study. HR < 1 implied improved overall survival in the experimental group. The size of each investigation represented the weighting factor (1/SE) assigned to the study.


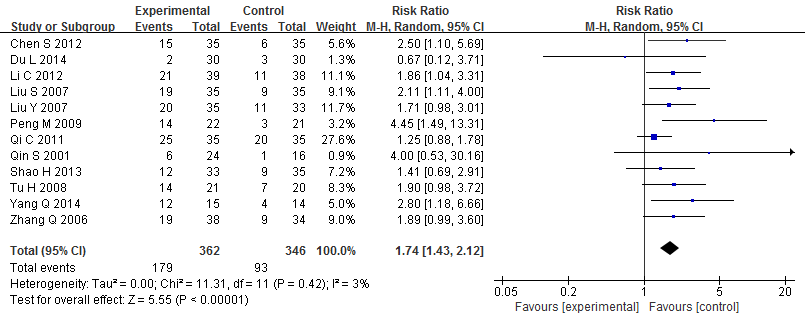


**Figure4: Forest plot of RR for evaluating of KPS in late-stage NSCLC patients in a random effects model.** The RR of KPS in the Rg3 and chemotherapy group was compared with that of the chemotherapy group. Individual studies are shown in the blue-colored squares, and the pooled datasets are shown by the diamond, representing the 95% conﬁdence interval (CI) of each study. RR > 1 implied a better quality of life in late-stage NSCLC patients among the experimental group. The size of each investigation represented the weighting factor (1/SE) assigned to the study.


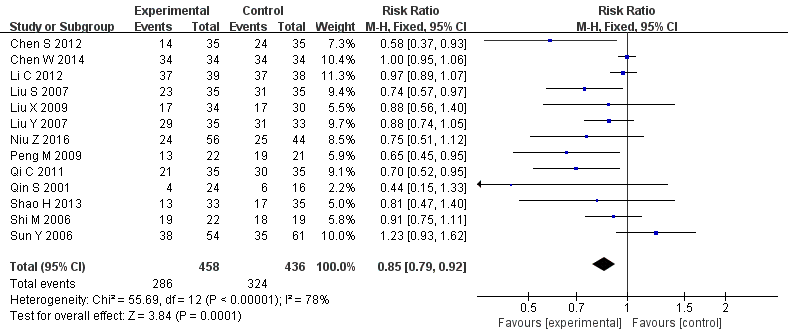


**Figure5: Forest plot of RR for evaluating of declination of leucocyte count Forest plot of RR for evaluating the decline in leucocyte count in fixed effects model.** The RR in the Rg3 and chemotherapy group was compared with the chemotherapy group. Individual studies are shown in the blue-colored squares, and the pooled datasets are shown by the diamond, representing the 95% conﬁdence interval (CI) of each study. RR < 1 implied a lower decline of leucocyte count in the experimental group. The size of each investigation represented the weighting factor (1/SE) assigned to the study.


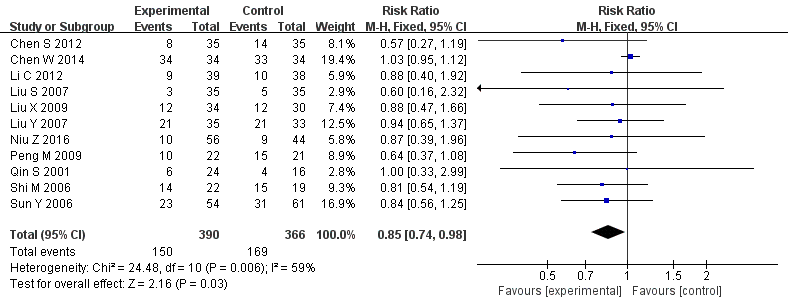


**Figure6: Forest plot of RR for evaluating of** **anemia in fixed effects model.** The RR in the Rg3 and chemotherapy group was compared with the chemotherapy group. Individual studies are shown in the blue-colored squares, and the pooled datasets are shown by the diamond, representing the 95% conﬁdence interval (CI) of each study. RR < 1 implied a lower risk of anemia in the experimental group. The size of each investigation represented the weighting factor (1/SE) assigned to the study.


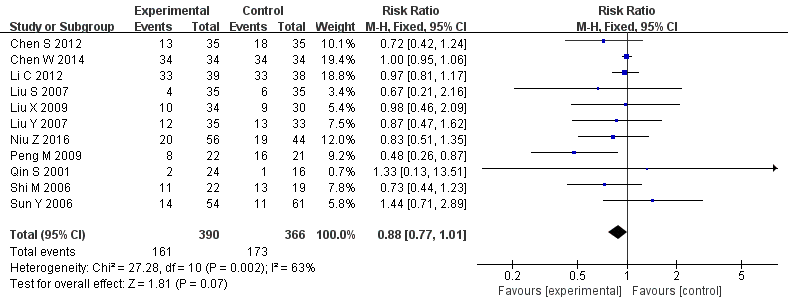


**Figure7:** **Forest plot of RR for evaluating of the declination of platelet count in fixed effects model.** The RR in the Rg3 and chemotherapy group was compared with the chemotherapy group. Individual studies are shown in the blue-colored squares, and the pooled datasets are shown by the diamond, representing the 95% conﬁdence interval (CI) of each study. The 95%CI between 0.77 and 1.01 implied no significant difference between two groups. The size of each investigation represented the weighting factor (1/SE) assigned to the study.


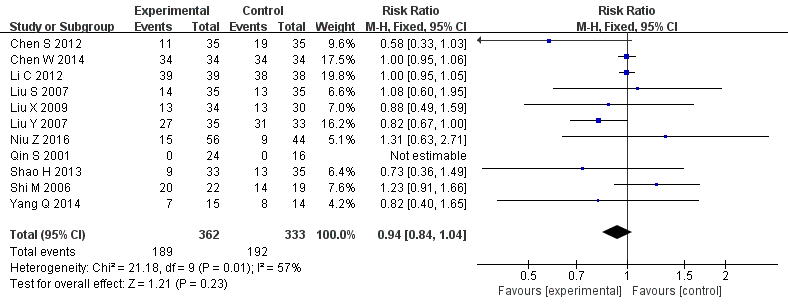


**Figure8:Forest plot of RR for evaluating of nausea and vomiting in fixed effects model.** The RR in the Rg3 and chemotherapy group was compared with the chemotherapy group. Individual studies are shown in the blue-colored squares, and the pooled datasets are shown by the diamond, representing the 95% conﬁdence interval (CI) of each study. The 95%CI between 0.84 and 1.04 implied no significant difference between two groups. The size of each investigation represented the weighting factor (1/SE) assigned to the study.


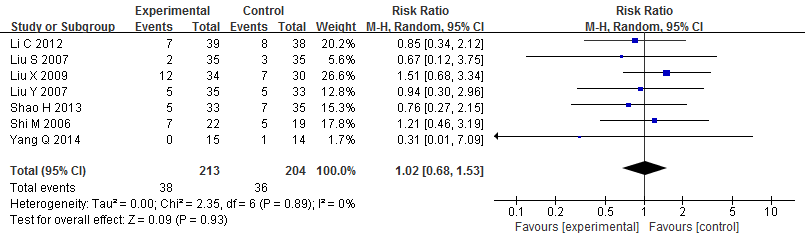


**Figure9:Forest plot of RR for evaluating of the hepatic dysfunction in random effects model.** The RR in the Rg3 and chemotherapy group was compared with the chemotherapy group. Individual studies are shown in the blue-colored squares, and the pooled datasets are shown by the diamond, representing the 95% conﬁdence interval (CI) of each study. The 95%CI between 0.68 and 1.53 implied no significant difference between two groups. The size of each investigation represented the weighting factor (1/SE) assigned to the study.


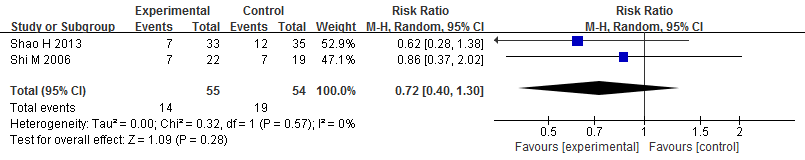


**Figure10: Forest plot of RR for evaluating of the constipine in random effects model.** The RR in the Rg3 and chemotherapy group was compared with the chemotherapy group. Individual studies are shown in the blue-colored squares, and the pooled datasets are shown by the diamond, representing the 95% conﬁdence interval (CI) of each study. The 95%CI between 0.72 and 1.30 implied no significant difference between two groups. The size of each investigation represented the weighting factor (1/SE) assigned to the study.


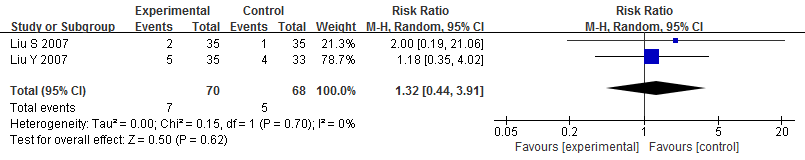


**Figure11: Forest plot of RR for evaluating of the peripheral nerve toxicity in random effects model.** The RR in the Rg3 and chemotherapy group was compared with the chemotherapy group. Individual studies are shown in the blue-colored squares, and the pooled datasets are shown by the diamond, representing the 95% conﬁdence interval (CI) of each study. The 95%CI between 0.44 and 3.91 implied no significant difference between two groups. The size of each investigation represented the weighting factor (1/SE) assigned to the study.


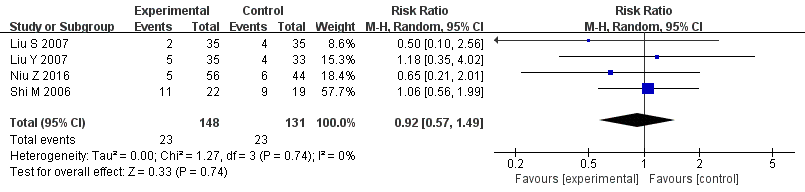


**Figure12: Forest plot of RR for evaluating of alopecia in random effects model.** The RR in the Rg3 and chemotherapy group was compared with the chemotherapy group. Individual studies are shown in the blue-colored squares, and the pooled datasets are shown by the diamond, representing the 95% conﬁdence interval (CI) of each study. The 95%CI between 0.57 and 1.49 implied no significant difference between two groups. The size of each investigation represented the weighting factor (1/SE) assigned to the study.


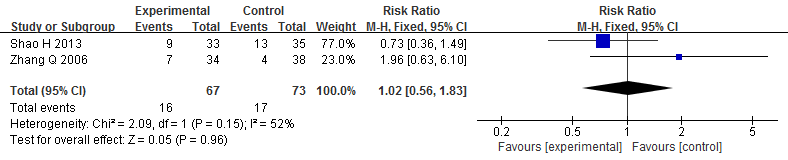


**Figure13: Forest plot of (Risk Ratio) RR for evaluating of fatigue in fixed effects model.** The RR in the Rg3 and chemotherapy group was compared with the chemotherapy group. Individual studies are shown in the blue-colored squares, and the pooled datasets are shown by the diamond, representing the 95% conﬁdence interval (CI) of each study. The 95%CI between 0.56 and 1.83 implied no significant difference between two groups. The size of each investigation represented the weighting factor (1/SE) assigned to the study.


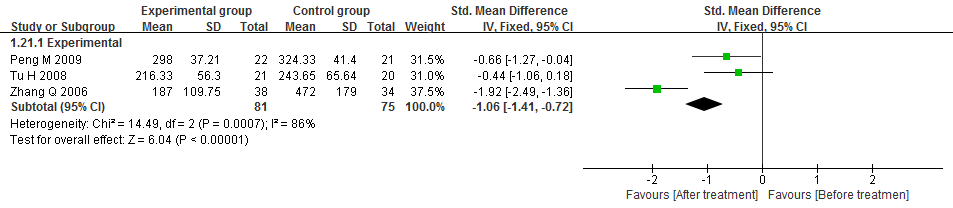


**Figure14: Forest plot of Std. Mean Difference for evaluating of expression of VEGF in peripheral blood of NSCLC patients between the periods of before and after treatment in fixed effect model.** The SMD of expression of VEGF in peripheral blood in the Rg3 and chemotherapy group was compared with the chemotherapy group. Individual studies are shown in the green-colored squares, and the pooled datasets are shown by the diamond, representing the 95% conﬁdence interval (CI) of each study. SMD <0 and P < 0.05 implied a lower expression of VEGF in the experimental group. The size of each investigation represented the weighting factor (1/SE) assigned to the study.


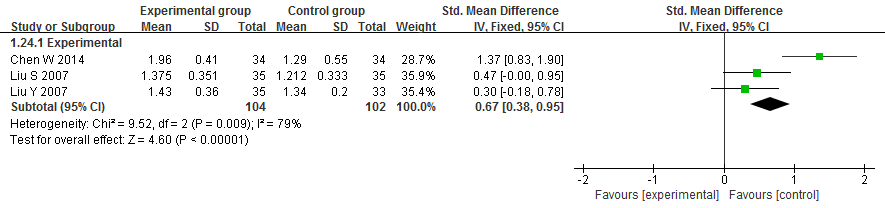


**Figure15: Forest plot of Std. Mean Difference for evaluating of the ratio of CD4/CD8 in peripheral blood of NSCLC patients between the periods of before and after treatment in fixed effect model.**The SMD of the ratio of CD4/CD8 in the peripheral blood in the Rg3 and chemotherapy group was compared with the chemotherapy group. Individual studies are shown in the green-colored squares, and the pooled datasets are shown by the diamonds, representing the 95% conﬁdence interval (CI) of each study. SMD ＞0 and P < 0.05 implied a more enhancement of the ratio of CD4/CD8 in experimental group. The size of each investigation represented the weighting factor (1/SE) assigned to the study.
