# Supplementary material for: Ginsenoside Rg3 Serves as an Adjuvant Chemotherapeutic Agent and VEGF Inhibitor in the Treatment of Non-Small Cell Lung Cancer: A Meta-Analysis and Systematic Review
Source: Evid Based Complement Alternat Med. 2016 Oct 5;2016:7826753. doi: 10.1155/2016/7826753 (PMC5069366; doi:10.1155/2016/7826753)
Supplement: Supplementary file 2 [file 7826753.f2.docx]

**Supplementary file2: Negative results
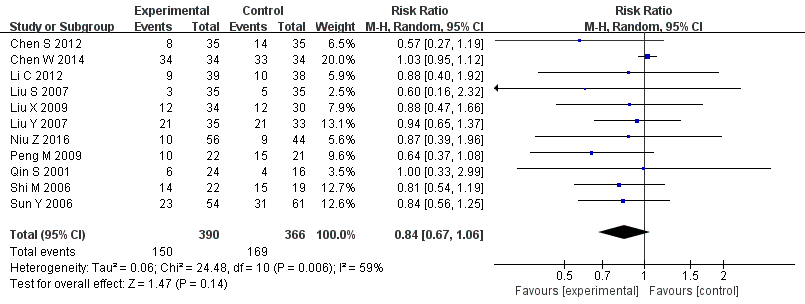
**

**Figure1: Forest plot of RR for evaluating of the** **anemia induced by chemotherapy in random effects model.** The RR of the anemia in Rg3 and chemotherapy group was compared with the chemotherapy group. Individual study is shown in the square with blue color, and the pooled datasets were shown in the diamond, representing the 95% conﬁdence interval (CI) of each study. The 95%CI between 0.67 and 1.06 implied no significant difference between two groups. The size of each investigation represented the weighting factor (1/SE) assigned to the study.


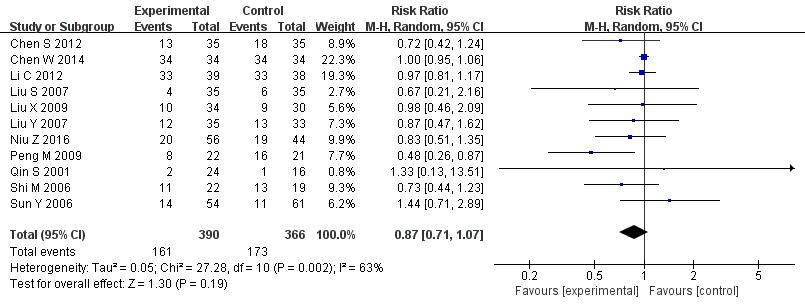


**Figure2: Forest plot of RR for evaluating of the declination of platelet count in random effects model.** The RR of the declination of platelet count in Rg3 and chemotherapy group was compared with the chemotherapy group. Individual study is shown in the square with blue color, and the pooled datasets were shown in the diamond, representing the 95% conﬁdence interval (CI) of each study. The 95%CI between 0.71 and 1.07 implied no significant difference between two groups. The size of each investigation represented the weighting factor (1/SE) assigned to the study.


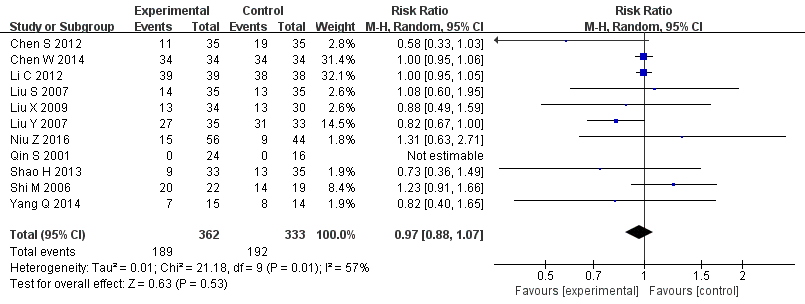


**Figure3: Forest plot of RR for evaluating of nausea and vomiting in random effects model.** The RR of nausea and vomiting in Rg3 and chemotherapy group was compared with the chemotherapy group. Individual study is shown in the square with blue color, and the pooled datasets were shown in the diamond, representing the 95% conﬁdence interval (CI) of each study. The 95%CI between 0.88 and 1.07 implied no significant difference between two groups. The size of each investigation represented the weighting factor (1/SE) assigned to the study.


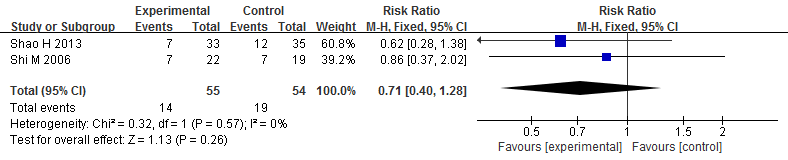


**Figure4: Forest plot of RR for evaluating of constipine in fixed effects model.** The RR of constipine in Rg3 and chemotherapy group was compared with the chemotherapy group. Individual study is shown in the square with blue color, and the pooled datasets were shown in the diamond, representing the 95% conﬁdence interval (CI) of each study. The 95%CI between 0.40 and 1.28 implied no significant difference between two groups. The size of each investigation represented the weighting factor (1/SE) assigned to the study.


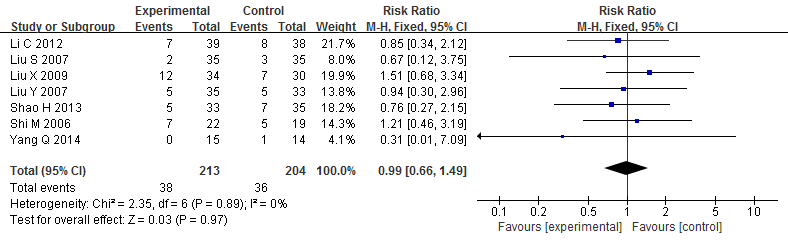


**Figure5: Forest plot of RR for evaluating of hepatic dysfunction in fixed effects model.** The RR of hepatic dysfunction in Rg3 and chemotherapy group was compared with the chemotherapy group. Individual study is shown in the square with blue color, and the pooled datasets were shown in the diamond, representing the 95% conﬁdence interval (CI) of each study. The 95%CI between 0.66 and 1.49 implied no significant difference between two groups. The size of each investigation represented the weighting factor (1/SE) assigned to the study.


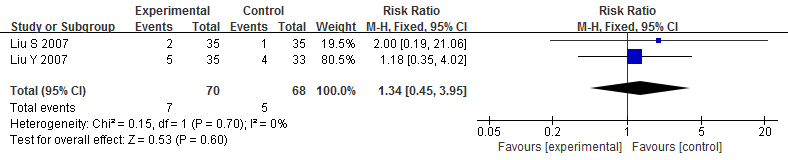


**Figure6: Forest plot of RR for evaluating of peripheral nerve toxicity in fixed effects model.** The RR of peripheral nerve toxicity in Rg3 and chemotherapy group was compared with the chemotherapy group. Individual study is shown in the square with blue color, and the pooled datasets were shown in the diamond, representing the 95% conﬁdence interval (CI) of each study. The 95%CI between 0.45 and 3.95 implied no significant difference between two groups. The size of each investigation represented the weighting factor (1/SE) assigned to the study.


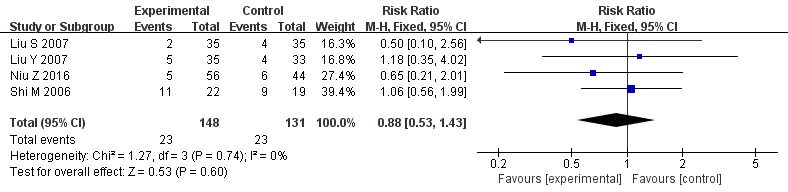


**Figure7: Forest plot of RR for evaluating of alopecia in fixed effects model.** The RR of alopecia in Rg3 and chemotherapy group was compared with the chemotherapy group. Individual study is shown in the square with blue color, and the pooled datasets were shown in the diamond, representing the 95% conﬁdence interval (CI) of each study. The 95%CI between 0.53 and 1.43 implied no significant difference between two groups. The size of each investigation represented the weighting factor (1/SE) assigned to the study.


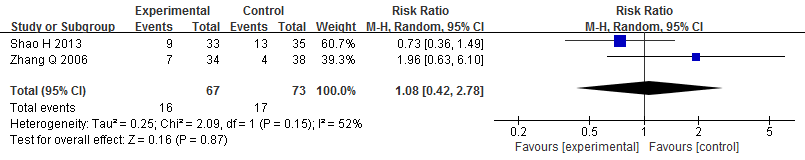


**Figure8: Forest plot of RR for evaluating of fatigue in random effects model.** The RR of fatigue in Rg3 and chemotherapy group was compared with the chemotherapy group. Individual study is shown in the square with blue color, and the pooled datasets were shown in the diamond, representing the 95% conﬁdence interval (CI) of each study. The 95%CI between 0.42 and 2.78 implied no significant difference between two groups. The size of each investigation represented the weighting factor (1/SE) assigned to the study.
