# Supplementary material for: Ginsenoside Rg3 Serves as an Adjuvant Chemotherapeutic Agent and VEGF Inhibitor in the Treatment of Non-Small Cell Lung Cancer: A Meta-Analysis and Systematic Review
Source: Evid Based Complement Alternat Med. 2016 Oct 5;2016:7826753. doi: 10.1155/2016/7826753 (PMC5069366; doi:10.1155/2016/7826753)
Supplement: Supplementary file 3 [file 7826753.f3.docx]

RR

Egger's test

------------------------------------------------------------------------------

Std_Eff | Coef. Std. Err. t P>|t| [95% Conf. Interval]

-------------+----------------------------------------------------------------

slope | 12.50446 6.381454 1.96 0.069 -1.097287 26.10621

bias | -26.88913 11.58187 -2.32 0.035 -51.5753 -2.202966

DCR

Egger's test

------------------------------------------------------------------------------

Std_Eff | Coef. Std. Err. t P>|t| [95% Conf. Interval]

-------------+----------------------------------------------------------------

slope | -.2621264 1.155282 -0.23 0.823 -2.711215 2.186962

bias | 1.465345 1.914075 0.77 0.455 -2.592314 5.523003

KPS

Egger's test

------------------------------------------------------------------------------

Std_Eff | Coef. Std. Err. t P>|t| [95% Conf. Interval]

-------------+----------------------------------------------------------------

slope | 10.99823 5.342421 2.06 0.067 -.9054293 22.90188

bias | -20.4273 8.960925 -2.28 0.046 -40.39348 -.4611115

Decline of leucocyte count

Egger's test

------------------------------------------------------------------------------

Std_Eff | Coef. Std. Err. t P>|t| [95% Conf. Interval]

-------------+----------------------------------------------------------------

slope | .3670656 .6369795 0.58 0.577 -1.052213 1.786345

bias | -.8658317 1.097439 -0.79 0.448 -3.311078 1.579414

Anemia

Egger's test

------------------------------------------------------------------------------

Std_Eff | Coef. Std. Err. t P>|t| [95% Conf. Interval]

-------------+----------------------------------------------------------------

slope | 4.814415 4.947009 0.97 0.359 -6.593409 16.22224

bias | -13.23726 9.066037 -1.46 0.182 -34.14358 7.669056

Decline of platelet count

Egger's test

------------------------------------------------------------------------------

Std_Eff | Coef. Std. Err. t P>|t| [95% Conf. Interval]

-------------+----------------------------------------------------------------

slope | 11.61472 5.545881 2.09 0.070 -1.174109 24.40354

bias | -26.18523 9.88103 -2.65 0.029 -48.97092 -3.399528

Nausea and vomiting

Egger's test

------------------------------------------------------------------------------

Std_Eff | Coef. Std. Err. t P>|t| [95% Conf. Interval]

-------------+----------------------------------------------------------------

slope | -4.525352 2.102528 -2.15 0.075 -9.670053 .6193497

bias | 5.913918 3.674474 1.61 0.159 -3.077195 14.90503

Hepatic dysfunction

Egger's test

------------------------------------------------------------------------------

Std_Eff | Coef. Std. Err. t P>|t| [95% Conf. Interval]

-------------+----------------------------------------------------------------

slope | 33.20803 11.40051 2.91 0.044 1.555154 64.86091

bias | -62.21635 17.36714 -3.58 0.023 -110.4353 -13.99744

Alopecia

Egger's test

------------------------------------------------------------------------------

Std_Eff | Coef. Std. Err. t P>|t| [95% Conf. Interval]

-------------+----------------------------------------------------------------

slope | 30.22163 28.55734 1.06 0.401 -92.65067 153.0939

bias | -58.95999 40.7144 -1.45 0.285 -234.1399 116.2199

HR

Egger's test

------------------------------------------------------------------------------

Std_Eff | Coef. Std. Err. t P>|t| [95% Conf. Interval]

-------------+----------------------------------------------------------------

slope | .1122058 .2037216 0.55 0.611 -.4534162 .6778277

bias | -2.125101 .9264078 -2.29 0.083 -4.697221 .4470196
